# Supplementary material for: Safety and immunogenicity of ChAd63-KH vaccine in post-kala-azar dermal leishmaniasis patients in Sudan
Source: Mol Ther. 2021 Mar 27;29(7):2366–77. doi: 10.1016/j.ymthe.2021.03.020 (PMC8261165; doi:10.1016/j.ymthe.2021.03.020)
Supplement: Document S1. Figures S1 and S2 [file mmc1.pdf]

YMTHE, Volume *29*

Supplemental Information

Safety and immunogenicity of ChAd63-KH vaccine in post-kala-azar dermal leishmaniasis patients in Sudan

Brima M. Younis, Mohamed Osman, Eltahir A.G. Khalil, Francesco Santoro, Simone Furini, Rebecca Wiggins, Ada Keding, Monica Carraro, Anas E.A. Musa, Mujahid A.A. Abdarahaman, Laura Mandefield, Martin Bland, Toni Aebischer, Rhian Gabe, Alison M. Layton, Charles J.N. Lacey, Paul M. Kaye, and Ahmed M. Musa

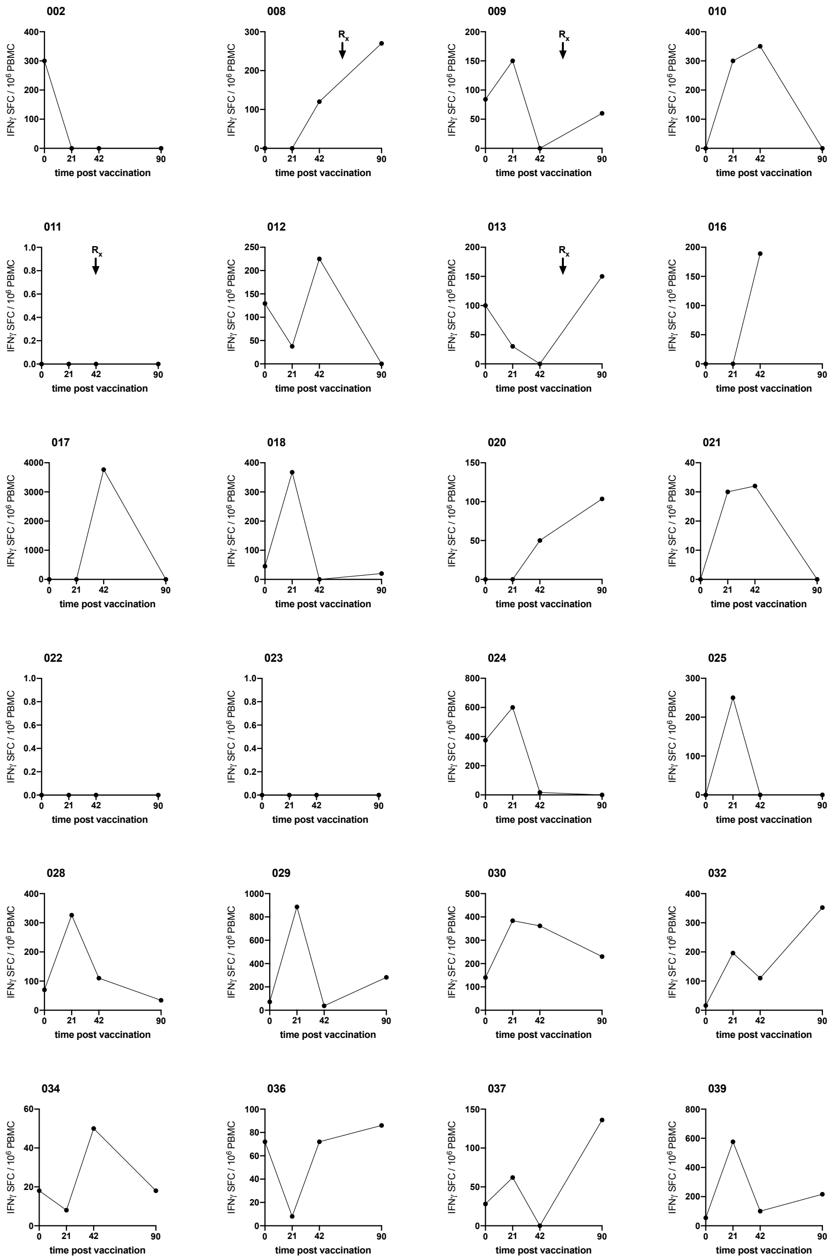

Figure S1. Kinetics of ELISpot response to KMP-11 peptide pool.

Data are shown for all individuals measured at d0, d21, d42 and d90 post vaccination from adult low dose (002, 008, 009, 010, 011, 012, 013, 016), adult high dose (017, 018, 020, 021, 022, 023, 024, 025) and adolescent high dose (028, 029, 030, 032, 034, 036, 037, 039) cohorts. Treatment was given to patients 008, 009, 011 and 013 prior to assessment of T cell response at d90 (Rx). Data are means of duplicate wells at each assay time point and have had medium only controls subtracted.

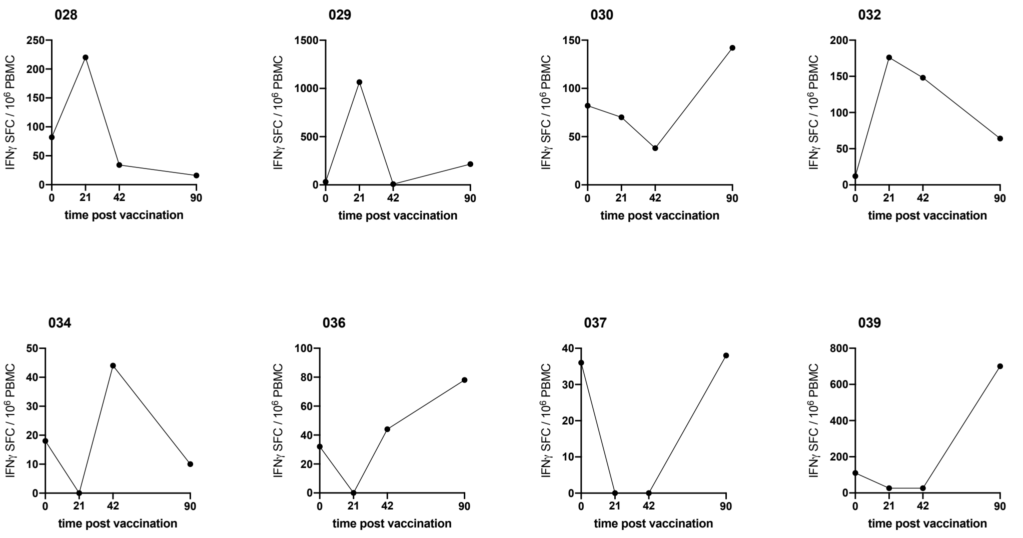

Figure S2. Kinetics of ELISpot response to HASPB N terminal peptide pool.

Data are shown for all individuals measured at d0, d21, d42 and d90 post vaccination from adolescent high dose (028, 029, 030, 032, 034, 036, 037, 039) cohort. Data are means of duplicate wells at each assay time point and have had medium only controls subtracted.

Supplemental Tables

Table S1. Demographic, baseline and AEs summary for study participants.

Sheet 1 provides demographic characteristics and biochemistry and haematology data at baseline. Sheet 2 summarises local and systemic AE number by cohort. Sheets 3 and 4 summarise AEs by relatedness to study intervention. Sheet 5 lists all reported AEs and grade.

Table S2. Transcriptomics analysis raw data

Sheet 1 contains count data for each patient at each time point examined. Sheet 2 contains patient descriptors and read depth and rate of target detection. Sheets 3 and 4 contain DE gene lists for adults (sheet 3) and adolescents (sheet 4) comparing those vaccinated with 7.5X1010 vp ChAd63-KH to pre-vaccination baseline.

Table S3. Module descriptions and results of machine learning.

Sheet 1 contains genes associated with each immune module used in this study.

Sheet 2 contains genes predicted to be associated with clinical response based on analysis using all modules or only modules DE at $p>0.05$.

Remaining sheets provide lists of the significantly enriched modules for each cohort at each time point and /or dose.

Table S4. Gene set enrichment analysis

Sheets contain output from EnrichR for GO Biological Processes 2018, Cellular Component 2018, Reactome 2016 and WikiPathways 2019 for DE genes at day 1 post vaccination in adult high dose and adolescent high dose cohorts.
